# Multi-model attribution of upper-ocean temperature changes using an isothermal approach

**DOI:** 10.1038/srep26926

**Published:** 2016-06-01

**Authors:** Evan Weller, Seung-Ki Min, Matthew D. Palmer, Donghyun Lee, Bo Young Yim, Sang-Wook Yeh

**Affiliations:** 1 School of Environmental Science and Engineering, Pohang University of Science and Technology, Pohang, Korea; 2 Met Office Hadley Centre, Exeter, UK; 3 Department of Marine Sciences and Convergent Technology, Hanyang University, ERICA, Korea

## Abstract

Both air-sea heat exchanges and changes in ocean advection have contributed to observed upper-ocean warming most evident in the late-twentieth century. However, it is predominantly via changes in air-sea heat fluxes that human-induced climate forcings, such as increasing greenhouse gases, and other natural factors such as volcanic aerosols, have influenced global ocean heat content. The present study builds on previous work using two different indicators of upper-ocean temperature changes for the detection of both anthropogenic and natural external climate forcings. Using simulations from phase 5 of the Coupled Model Intercomparison Project, we compare mean temperatures above a fixed isotherm with the more widely adopted approach of using a fixed depth. We present the first multi-model ensemble detection and attribution analysis using the fixed isotherm approach to robustly detect both anthropogenic and natural external influences on upper-ocean temperatures. Although contributions from multidecadal natural variability cannot be fully removed, both the large multi-model ensemble size and properties of the isotherm analysis reduce internal variability of the ocean, resulting in better observation-model comparison of temperature changes since the 1950s. We further show that the high temporal resolution afforded by the isotherm analysis is required to detect natural external influences such as volcanic cooling events in the upper-ocean because the radiative effect of volcanic forcings is short-lived.

The degree to which ocean temperatures have warmed in recent decades varies considerably with depth and ocean basins, but overall the upper-ocean (i.e. ~200 m) has warmed at a much higher rate^1^,^2^,^3^,^4^,^5^,^6^,^7^. In addition, underneath the excessive warming some regions have displayed significant cooling at thermocline and sub-thermocline levels^2^,^8^. For the upper-ocean, differences in regional warming rates can arise in part due to changes in two key drivers, surface forcing such as air-sea heat fluxes, and ocean advection of the near-surface waters^9^,^10^. There is a need to consider upper-ocean warming in the sense of separating surface heat fluxes and ocean advection responses. This is because the atmosphere and land have very minor heat capacity on multi-annual timescales, therefore the ocean must be the recipient of net radiative budget changes via air-sea heat exchange from increasing anthropogenic greenhouse gases and changes in natural external climatic forcings such as volcanic activities^11^,^12^. Anthropogenic emissions of aerosols have recently also been shown to be important for fluctuations in past regional upper ocean temperatures^13^.

A number of studies find natural external climatic forcing signals (i.e. solar and volcanic activities) indistinguishable from intrinsic natural internal ocean variability^2^,^4^,^6^. However, these findings often only consider external influences over longer periods, for example pentad or decadal averaging timescales, of which major volcanic aerosol forcings do not completely persist in the upper-ocean^14^. Indeed, it has been shown in a single model study that using a higher temporal resolution (i.e. 2-year timescale) and a more suitable upper-ocean temperature estimate, the influence of such natural external forcings can be detected^11^. That is, by quantifying upper-ocean temperature changes relative to a fixed isotherm, rather than the more commonly adopted method of a fixed depth^3^,^5^,^7^, noise associated with intrinsic internal variability in the oceans is dramatically reduced for both observations and models, allowing the contribution from surface heat fluxes alone, to be better isolated^9^,^11^,^15^. However these conclusions are based on analysis of only a single model^11^. Therefore this issue would benefit from further analysis including an extended observation length, updated observational data sets including recent work on bias corrections, but more importantly strengthened by taking advantage of the large state-of-the-art multi-model ensemble from the latest generation of climate models.

In the context of attributing contributions from different external forcings to upper-ocean temperature changes, we use a suite of Coupled Model Intercomparison Project phase 5 (CMIP5)^16^ models (Table S1) forced with all historical radiative forcings (ALL) as well as individual forcing runs of natural external influences (solar and volcanic activities, denoted NAT) (see Methods for details). We calculate the overall anthropogenic forcing (greenhouse gas, sulphate aerosol, and ozone changes, denoted ANT) as the difference between ALL and NAT. We use these simulations to (i) compare upper-ocean temperature changes estimated relative to a fixed isotherm (average temperature above the 14 °C isotherm, denoted T_14°C_) versus a fixed depth (average temperature in the upper 220 m, denoted T_220m_); and (ii) examine whether the upper-ocean temperature trends from the two methods can be attributed to external anthropogenic and/or natural external climatic forcings. The 14 °C isotherm and fixed depth of 220 m are used to calculate the two upper-ocean temperature estimates to allow direct comparison with previous studies^9^,^10^,^11^. The 14 °C isotherm is suggested to provide good spatial coverage of the upper-ocean, at low to mid-latitudes, throughout the observational record, and 220 m because it is the overall time-spatial mean depth of the 14 °C isotherm in low and mid-latitudes^9^.

## Results

### Observed and CMIP5 simulated upper-ocean temperatures

**Figure 1:**
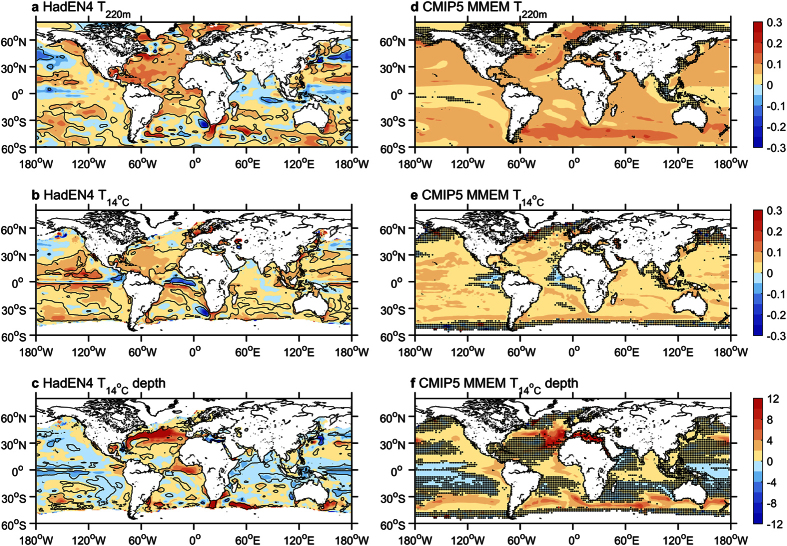
Spatial trends of observed and simulated upper-ocean temperatures. (**a**) Trend (°C decade^−1^) of average temperature anomaly above 220 m (T_220m_) from HadEN4 over 1951–2010. Contours enclose trends significant at the 95% level. (**b**) The same as (**a**) but for the average temperature anomaly above the 14 °C isotherm (T_14°C_). (**c**) The same as (**a**) but for depth trends (m decade^−1^) of the 14 °C isotherm (D_14°C_). (**d**–**f**) The same as (**a**–**c**) but for the mean trends of the ALL forcing CMIP5 multi-model ensemble. Stippling indicates where less than 15 models (out of 22, ~70%) agree on the sign. All maps were produced using licensed MATLAB (release R2015B available at http://au.mathworks.com/products/matlab/index.html).

Figure 1a,b compare the spatial maps of trends in T_220m_ and T_14°C_, respectively, over the period 1951–2010 from HadEN4 observations^17^. We show only results from spatially complete infilled estimates from HadEN4, but note they are very similar to sub-sampled spatial patterns^11^, but spatially less variable. In addition, we consider only results for HadEN4 because generally the ensemble spread amongst observational products is small compared with the anthropogenic signal^18^, and HadEN4 estimates are improved relative to previous versions^17^. Averaged over the upper 220 m, the largest widespread increase in temperature has occurred in the North Atlantic, at a rate about twice that of other basins, of which has been documented in previous studies^5^,^7^,^10^,^19^,^20^ (Fig. 1a). The largest regions with decreasing trends in T_220m_ occur along the equator and in the north of the Pacific and Indian Oceans. In contrast, for temperatures averaged above the 14 °C isotherm, the warming in the North Atlantic is not as striking, and there is cooling along the equator in the Atlantic (Fig. 1b). Similarly, decreasing temperature trends in the Pacific are not as marked for T_14°C_ as they are for T_220m_, and are almost absent in the Indian Ocean. The cooling in the equatorial Pacific is much more confined in T_14°C,_ with large warming either side of the equator. Decreasing trends in upper-ocean temperatures from both analyses tend to be associated with a shoaling of the 14 °C isotherm, whereas the large temperature increase in the North Atlantic is associated with a strong deepening (Fig. 1c).

**Figure 2:**
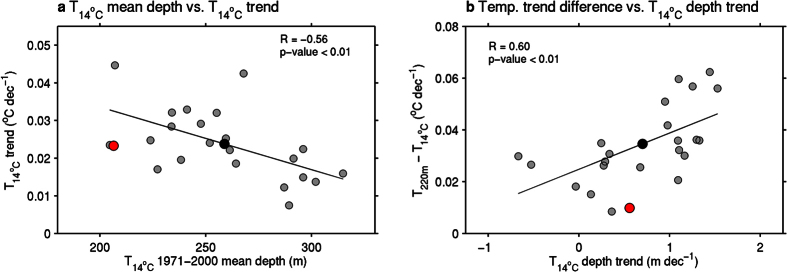
Inter-model relationships associated with global-mean upper-ocean temperature changes. (**a**) Scatterplot of the global-mean climatological (1971–2000) depth of the 14 °C isotherm in the ALL forcing model simulations (grey) and observations (red) versus the trend in average temperature anomalies above the 14 °C isotherm (T_14°C_). Also shown is the multi-model ensemble mean (black). (**b**) The same as (**a**) but for the global-mean trend in the depth of T_14°C_ versus difference between the trends in average ocean temperature anomalies above 220 m (T_220m_) minus T_14°C_. The line-of-best-fit, R and p-values are also shown for both relationships, where the regression lines are both statistically significant at the 99% confidence level.

Equivalent maps calculated from the ALL forcing CMIP5 multi-model ensemble mean (MMEM) are shown in Fig. 1d–f. For T_220m_, the MMEM exhibit no decreasing trends as seen in the observations, and the increasing trends are generally stronger, except for the North Atlantic (Fig. 1a,d). However, regions that display weak increasing trends do in fact coincide with the regions of decreasing trends in the observations, for example, along the equator in the Pacific and the eastern Indian Ocean. For T_14°C_, the MMEM is in good agreement with observations, and in some regions weak decreasing trends are simulated, consistent with the observed map (Fig. 1b,e). This is evident over the far eastern equatorial regions of the Pacific and Atlantic Oceans, and appears to be related to a deepening of the isotherm (Fig. 1c,f). We note that some differences between the observations and the MMEM appear to arise from large-scale modes of intrinsic internal variability, of which the MMEM does not capture. For example, in the Pacific, the observed temperature trend patterns somewhat resemble the pattern often associated with the Pacific Interdecadal Oscillation^21^. Using the isotherm approach appears to remove some of this large-scale noise from the observations (*c.f*. Fig. 1a,b) resulting in the better agreement with the MMEM, yet it cannot be assumed that the influence of such internal climate variability via all processes (i.e. all upper-to-deeper vertical transport^22^,^23^ and horizontal re-distribution^24^ of heat) can be removed completely. Therefore, a caveat in the detection and attribution of external forcings in upper-ocean temperature trends will be that variability of this kind is a potential source of observation-model inconsistency. This will be discussed further in following sections. Comparison of trends in the 14 °C isotherm depth between observations and the MMEM also indicates spatial consistency, except for an eastward displacement of the large increase in the North Atlantic and a weak deepening in the tropical Indian Ocean in the MMEM (Fig. 1c,f). These small differences are related to similar spatial biases in the MMEM mean state compared to the observations (Fig. S1a,c). It is found that the MMEM simulates a global-mean depth of the 14 °C isotherm depth that is slightly deeper than observations, yet with a comparable global-mean T_14°C_ trend (Fig. 2a). Examining individual models, a small number exhibit a large difference compared to the observed depth of the 14 °C isotherm, of up to approximately 100 m, which could be related to a mean state bias or the model resolution. Further, a significant inter-model relationship exists between the time-spatial mean depth of the 14 °C isotherm and the trend in T_14°C_ (Fig. 2a). This is as one might expect because the warming signal is surface-intensified in the oceans^2^,^6^, so those models with a shallower 14 °C isotherm tend to show a larger rate of warming, with mean temperature and isotherm changes making separable and additive contributions to such changes in T_14°C_^10^.

Overall, consistent with previous studies^9^,^11^, the T_14°C_ trends from observations, and the CMIP5 MMEM, have more spatial uniformity, but also a reduced variance in model ensemble trend spread, compared to T_220m_ (Fig. S2). This results from removing some of the ocean circulation influences on T_14°C_ that are associated with intrinsic internal variability, and plays a large role in fixed depth temperature trends^11^. This is further supported by the fact that the spatial differences between T_220m_ and T_14°C_ trends for both observations and the MMEM are similar to the overall spatial pattern of trends in the depth of the 14 °C isotherm (*c.f*. Fig. 1c,f and Fig. S1b,d). Figure 2b highlights the statistically significant inter-model relationship that exists between the global-mean differences between T_220m_ and T_14°C_ and trends in the depth of the 14 °C isotherm. The MMEM has a larger difference between the two temperature trends (T_220m_ minus T_14°C_) despite a similar global-mean depth trend compared to the observations. More so, models with a larger depth trend in the 14 °C isotherm show a larger discrepancy between T_220m_ and T_14°C_ trends. This is due to both changes in temperature above the 14 °C isotherm and the depth of the 14 °C isotherm contributing to the temperature changes in the upper 220 m ocean layer^10^.

**Figure 3:**
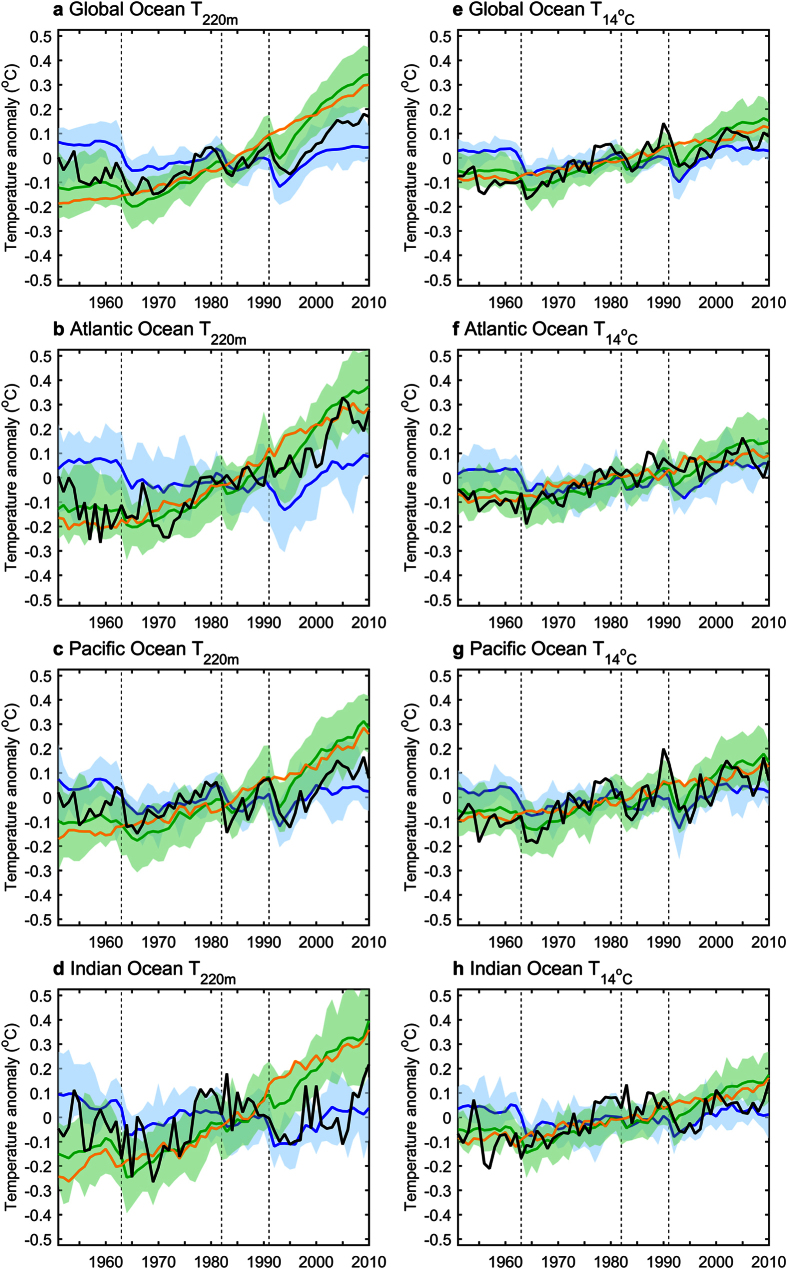
Observed and simulated upper-ocean temperature anomalies averaged over the oceans basins. (**a**) Annual-mean average ocean temperature anomalies (referenced to 1951–2010) above 220 m (T_220m_) for the Global Ocean. HadEN4 (black) is compared to the CMIP5 multi-model ensemble response for all (ALL, green), natural (NAT, blue) and anthropogenic (ANT, estimated as ALL minus NAT, orange) forcing simulations. Thick lines indicate multi-model means, and shading the 5–95% spread of ALL (green) and NAT (blue) ensembles. (**b**–**d**), The same as (**a**) but for the Atlantic, Pacific and Indian Oceans basins. (**e**–**h**) The same as (**a**–**d**) but for annual-mean average temperature anomalies above the 14 °C isotherm (T_14°C_). In all panels, vertical lines indicate approximate timing of the major volcanic eruptions. For consistency, average temperatures are calculated for regions that have data for both T_220m_ and T_14°C_.

Time series of the annual global-mean T_220m_ anomalies show that the ALL forcing MMEM simulates much stronger warming compared to the observations from the early 1990’s (Fig. 3a). This may be attributable to (i) less cooling response and a quicker recovery associated with the large 1991 volcanic eruption and a lack of simulating a series of recent small tropical volcanic eruptions^25^; (ii) intrinsic internal multidecadal climate variability^25^,^26^,^27^; or (iii) the fact that historical data are very sparse in many ocean regions especially in the early period^9^,^28^, hence the long-term warming trend might be underestimated in the observations. The main regional contributions to the excessive warming in the global-mean T_220m_ arises from stronger warming in the Pacific and Indian Oceans in the MMEM, which are considerably less sampled compared to the Atlantic (Fig. 3b–d). In the NAT simulations, responses due to the major volcanic eruptions (i.e. 1963, 1982 and 1991) are clearly evident and have a significant contribution to the ALL simulations variability (Fig. 3a–d). In addition the NAT forcing induces a short positive contribution during the 1950s, yet there is no significant long-term trend to explain the large warming in the observations.

On the other hand, the ALL forcing simulations of T_14°C_ show considerable observation-model improvement compared to T_220m_ in all basins (Fig. 3e–h). Overall, the root-mean-square (RMS) values associated with the observed T_14°C_ time series are lower than those for T_220m_ except for the Pacific Ocean, and RMS values associated with the MMEM T_14°C_ time series are about half of those for T_220m_ (Table S2). The abrupt volcanic induced cooling events are again evident in observations and both the ALL and NAT simulations, but the long-term warming is more steady for the entire period compared to that from T_220m_. Similar to the larger qualitative agreement with observations for long-term trends in T_14°C_ compared to T_220m_, the RMS differences of the time series of T_14°C_ are also reduced between observations and the MMEM compared to T_220m_, by 29–57% for individual basins (Table S3). This reduction is slightly higher than previously reported using an ensemble from a single model (reduction of 15–40%)^11^. The largest reduction of the RMS differences occurs in the Indian Ocean, where the observations are dominated by strong sub-thermocline cooling which occurs closer to the surface than in the models^8^ (Fig. 1a,d).

### Optimal detection analysis

To detect contributions from anthropogenic (ANT) and natural external (NAT) influences on long-term variations in T_220m_ and T_14°C_, we employ an optimal fingerprinting technique^29^. In this method, observations are regressed via generalized linear regression onto the two multi-model simulated signals of ANT and NAT simultaneously (see Methods for details), in order to estimate the contribution by both anthropogenic and natural external forcings to changes in upper-ocean temperatures. Using a large set of unforced control simulations, we obtain an estimate of the internal climate variability, in addition to conducting a residual consistency test^29^ to compare model-simulated internal variability with observations. Resulting best estimates and uncertainty ranges (5–95% confidence intervals) of the scaling factors, which scale estimates of the responses to individual combinations of forcings to best reproduce observed changes, are used to determine whether external forcing fingerprints can be identified in the observations. The influence of external forcing is detected when a scaling factor is significantly greater than zero, and considered consistent with observations when its uncertainty ranges include unity.

**Figure 4:**
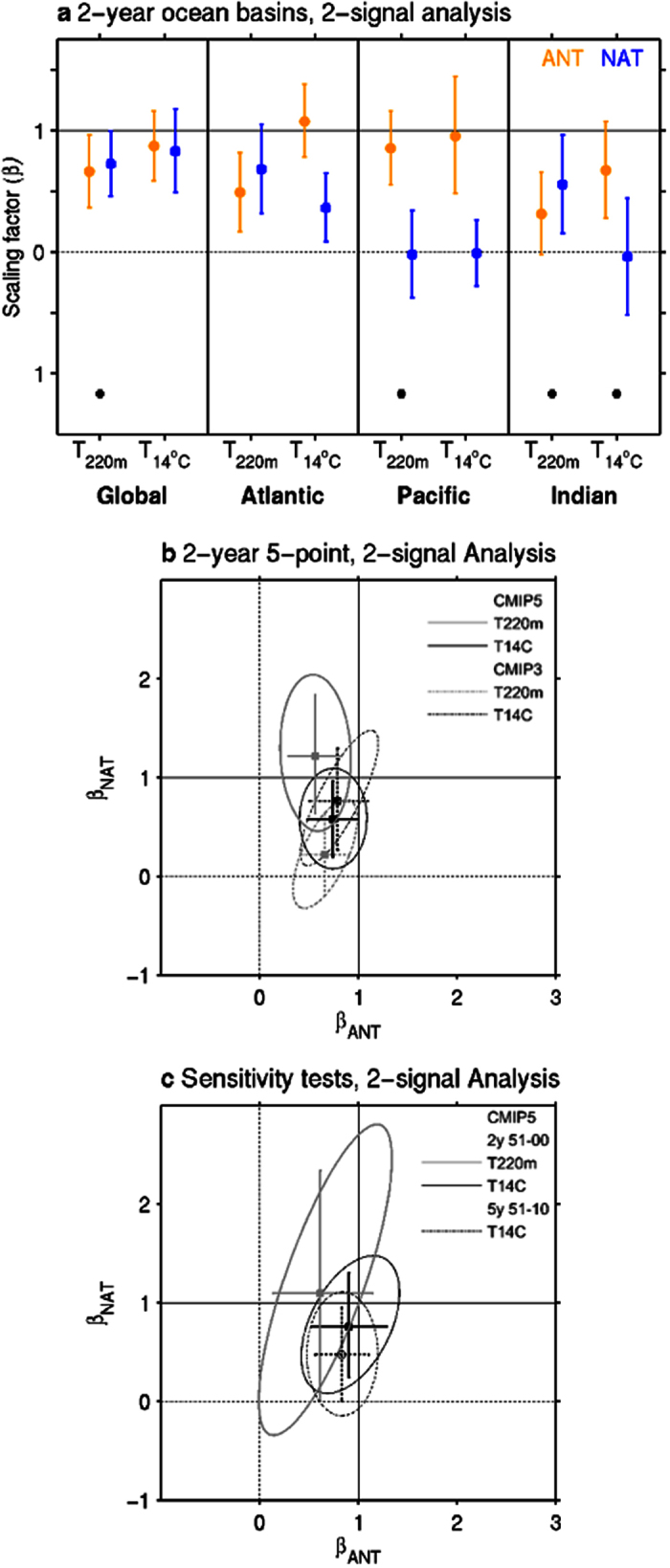
Results from optimal detection analysis of upper-ocean temperatures anomalies. (**a**) Estimated scaling factors for two-signals of anthropogenic (ANT) and natural (NAT) forcings by which the simulated 2-year averaged T_220m_ and T_14°C_ responses in the oceans should be multiplied to best match the observations over 1951–2010. Best estimates (data points) and 5–95% confidence intervals (error bars) of the scaling factors are displayed. Black squares under scaling factor pairs indicate that the residual variability remaining in the observations after removing the scaled response is inconsistent with model internal variability. (**b**) Estimated scaling factors for two-signals of ANT (x-axis) and NAT (y-axis) forcings based on a five-point spatial model fingerprint, composed of the average T_220m_ (gray) and T_14°C_ (black) responses in the North Atlantic, South Atlantic, North Pacific, South Pacific and Indian Oceans over 1951–2010 from CMIP5 (solid) and CMIP3 (dashed) ensembles, using an EOF truncation of 30. The 5–95% joint confidence for two-signals are represented by ellipses. (**c**) The same as (**b**) but using CMIP5 for two sensitivity tests, 1) repeating the analysis over the period 1951–2000 (solid), and 2) repeating the analysis using lower temporal resolution (i.e. 5-year time series, dashed). In all panels, a detectable response to an individual forcing occurs when scaling factors are significantly greater than zero. Consistency between observed and simulated responses is determined when scaling factors are not significantly different from unity.

We first conduct the fingerprint analysis using 2-year averaged global-mean and individual ocean basin T_220m_ and T_14°C_ time series over the period 1951–2010 (Fig. 4a). Scaling factors are shown which consider full empirical orthogonal function (EOF) dimensions (i.e. a truncation value 30 from a 2-year averaged 60-year time series). Using the 2-year temporal resolution removes some high frequency noise that we are not concerned with, yet still allows the quantification of larger influences from natural external forcing such as volcanic activities. Consistent with the qualitative analysis of the spatial and time series results, we find that the models overestimate the response to ANT for T_220m_ in all basins, but in the Pacific the 90% confidence interval includes unity (Fig. 4a). Interestingly, the model fingerprints also indicate an overestimation of the response to NAT or no detection. However, we note that the residual consistency test fails in all T_220m_ cases except the Atlantic, indicating that the residual variability that remains in the observations after removing the scaled response is inconsistent with model internal variability. Therefore, it is only in the Atlantic that both ANT and NAT forcing signals are robustly detected for T_220m_, owing to the larger warming signal over variability noise. For T_14°C_, the ANT response is robustly detected, and the best estimates lie close to unity, in all basins except the Indian Ocean (Fig. 4a). This indicates the models simulate well the long-term evolution of the observed T_14°C_ during 1951–2010. The residual consistency test is not passed for T_14°C_ in the Indian Ocean, which may be a result from both the mean bias of the 14 °C isotherm in the models, and the depth at which the excessive cooling is simulated in the models during the last fifty years^8^. A clear detection of a NAT signal for T_14°C_ is also shown for the global-mean and Atlantic analyses. The confidence interval associated with the global-mean includes unity, suggesting that the models upper-ocean heat content response to solar and volcanic changes is consistent with the observations.

Next we apply a five-point spatial model fingerprint analysis to the CMIP5 multi-model ensemble previously conducted using a single-model ensemble^11^. For example, we concatenate, by ocean basins which are also separated by hemisphere, the 2-year averaged temperature anomaly in the North Atlantic, South Atlantic, North Pacific, South Pacific and Indian Oceans, and repeat the two-signal fingerprint analysis over the period 1951–2010 (Fig. S3). The results are also compared to with those from a CMIP3^30^ multi-model ensemble to assess the robustness of the method used to estimate individual forcing contributions (see Methods for details). Figure 4b shows the resulting best estimate scaling factors and confidence intervals for the two generations of models, for an EOF truncation of 30 as in the individual basin analysis. Interestingly, both ANT and NAT fingerprints are detected in CMIP5 for T_220m_ and T_14°C_. However, we note that it is only for T_14°C_ that the detection results are robust, because the residual variability remaining in the observations for T_220m_, after removing the scaled response, is inconsistent with model internal variability (Fig. S4). For CMIP3, only ANT and NAT fingerprints are robustly detected for T_14°C_, with a best estimate scaling factor very close to CMIP5. For T_220m_ there is no detectable signal from NAT forcing for CMIP3, consistent with previous studies^11^. The difference between CMIP5 and CMIP3 could be due to more sophisticated aerosol schemes in the CMIP5 models which are better able to capture the external climatic forcing.

Overall, results from the five-point spatial model fingerprint confirms the ANT best estimate response in both T_220m_ and T_14°C_ is slightly overestimated in the models. Yet confidence intervals include unity for T_14°C_, and the ANT signal also needs to be scaled down more for T_220m_. Using a fixed depth of 260 m (the MMEM depth of the 14 °C isotherm (Fig. 2a) yields similar results. Our results differ from that of using a single-model ensemble^11^ over a slightly shorter period (i.e. 1950–1999), as we detect external signals for T_220m_ in the CMIP5 and CMIP3 multi-model ensembles. Here substantially increasing the number of noise samples in our analysis may aid in improving the signal-to-noise ratio over a longer period compared to a single-model analysis. Repeating the analysis over the shorter period 1951–2000, the same as in previous analyses^11^, we indeed obtain consistent results with such single-model ensemble analysis (Fig. 4c). For example, neither ANT nor NAT fingerprints are robustly detected for T_220m_ considering the joint confidence intervals (ellipses), yet we note the ANT signal by itself is, although overestimated, robustly detected (Fig. 4c).

Another sensitivity test we consider is the effect of temporal resolution to the fingerprint identification. Many studies report that the NAT signal is unable to be detected above intrinsic internal variability in upper-ocean temperatures^2^,^4^,^6^. However these studies considered longer time averaging scales to remove the majority of internal variability (i.e. 5 or 10-year temporal resolutions). However, such temporal resolutions also potentially remove the upper-oceans response to natural external forcings such as volcanic cooling events. This appears to be true, as by using the same CMIP5 MMEM and observations over the period 1951–2010 but applying a 5-year averaging, the NAT signal in T_14°C_ is no longer robustly detected (Fig. 4c). Hence, this result provides evidence for the need to consider a similar temporal resolution (i.e. 2-year rather than 5 or 10-year) to the radiative effect of volcanic eruptions, which is generally short-lived in the upper-ocean, about 1–3 years^14^, to capture any natural external forcing signal. This is different to the deep ocean layers, where volcanic aerosol influence has been shown to persist for decades after the event^14^,^31^. The role of ocean advection captured for each temporal resolution may also differ.

## Discussion and Summary

Building on previous work^9^,^11^, we strengthen detection and attribution conclusions on the influence of externally forced signals of anthropogenic influences, but more importantly also from natural external changes, simultaneously on observed upper-ocean temperatures. We present the first multi-model detection study employing isotherm diagnostics of subsurface ocean temperature changes for the past sixty years. As air-sea heat flux changes are the main mechanism by which the anthropogenically-induced and natural external factors influence upper-ocean temperature changes, using a fixed isotherm as an alternative to a fixed depth for analyses aids to better isolate the surface forcing from changes due to ocean dynamical changes. The combination of incomplete observations and changes in ocean advection can potentially confound our efforts in accurately estimating the rate of global ocean heat uptake. However, using an isothermal approach, we improve the signal-to-noise ratio, where noise refers to both small-scale phenomena, such as internal waves and eddies, but also presumably a proportion of large-scale coherent climate variability, such as that associated with major climate modes. The isotherm framework helps to remove some of the large-scale noise from the observations (i.e. the observed phase of climate modes that are generally of a different phase in climate model simulations). Overall, the reduction of both high and low frequency intrinsic variability in temperature above the 14 °C isotherm (T_14°C_) allows a more uniform trend to emerge^9^. Here, using a much larger ensemble of forced and unforced simulations from the latest generation of climate models and a fixed isotherm, we obtain a significant identification of both ANT and NAT fingerprints in the observed upper-ocean temperatures changes. This confirms previous results using only a single-model ensemble^11^. It is shown that the externally forced signals for the fixed isotherm analysis (T_14°C_) are not affected by the use of an ensemble of an earlier generation of models (i.e. CMIP3 compared to CMIP5), however the NAT signal is not detected in the fixed depth analysis of CMIP3 (i.e. T_220m_). The availability of individual forcing simulations may be one reason for the different detection results (i.e. linear additive calculations of ANT and NAT, see Methods for details) or the fact that the larger ensemble size for CMIP5 compared to CMIP3 enhances the positive identification of the NAT signal, as is the case when compared to a single-model analysis^11^, and the CMIP5 models contain more sophisticated aerosol schemes. Spatial trend maps and results from the optimal fingerprint analysis highlight the fact that the CMIP5 models tend to overestimate the upper-ocean warming during the last sixty years. However in poorly sampled regions, such as the Southern Hemisphere, it has been revealed that the observed changes are significantly underestimated^28^, leading to such differences with the CMIP5 MMEM.

Another factor of the observations not considered here, are contributions by decadal changes in modes of intrinsic internal variability. This may have partly contributed to the overestimation of the MMEM, as a slowdown in recent warming has been observed since the late 1990s^26^, partly driven by a large change in atmospheric circulations such as strengthening of the Walker circulation over the Pacific Ocean region^32^,^33^. Interestingly, trends of the upper-ocean time series (both T_220m_ and T_14°C_) after removal of the influence of the leading mode of the Pacific multidecadal variability (i.e. the Pacific Interdecadal Oscillation, Fig. S5), based on linear regression, reveal that such variability over the 60-year period investigated here has compounded anthropogenic influences (Table S4), despite the recent slowdown. We also note that although some influence of this variability appears to be reduced in the spatial patterns (Fig. 1a–d), similar small contributions due to the Pacific Interdecadal Oscillation are removed from both T_220m_ and T_14°C_ time series (Fig. S5). This question needs to be further investigated in future work through comprehensive assessments of both observed and modelled multidecadal variability modes and their connections to the upper ocean temperature variations. In contrast, a recent study on extreme temperature detection found better agreement between observed and modeled trends after removing internal climate variability influences on the observations^34^. However, in that case models underestimate the observed warming, resulting in improvement, yet here the model overestimation may simply suggest a systematic bias of the models, perhaps too sensitive, in the responses of upper ocean temperature to external forcing, causes of which remain uncertain.

Using an average temperature above a fixed isotherm as opposed to a fixed depth yields trends in better agreement with the observations. Therefore, some overestimation of the upper-ocean warming may also arise from a stronger influence on upper-ocean temperatures from ocean dynamical changes in the models. One possibility to examine this is by adopting an alternative approach to ocean heat content changes, whereby it is estimated relative to a fixed isotherm^10^, rather than a fixed depth, similar to the method used in the present study. Such a method allows the quantification of the separate contributions to heat content changes from the deepening of the reference isotherm, indicative of changes in ocean dynamics and circulation, versus warming above the isotherm, indicative of changes in surface air-sea heat fluxes. This method has previously been used to assess the individual contributions to observed changes in upper-ocean heat content^10^. Applying this method to a large, multi-model ensemble as used in the present study, would improve our understanding and allow quantification of the individual contributions to upper-ocean temperature changes, and help to address the concern of large uncertainty in the upper ocean heat content estimates^35^. This will be addressed in further work that is currently underway.

## Methods

### Ocean temperature data

#### HadEN4

The observations used are the Met Office Hadley Centre EN4 version 4.1.1 quality-controlled objective analysed subsurface ocean temperature^17^ (available from www.hadobs.metoffice.com/en4/). This dataset includes bias corrections^36^ to account for observational sampling techniques in past decades. It was previously noted that T_14°C_ measurements are insensitive to these correction^9^. We also find that using HadEN4 with another set of bias corrections^37^ do not change our results. The product has 1° × 1° spatial resolution and monthly temporal resolution.

### Model simulations

#### CMIP5 historical and RCP runs

We utilise both historical and RCP model experiments from the CMIP5 archive^16^. These include a single historical (1951–2005) and future greenhouse-gases under emission scenario of Representative Concentration Pathway (RCP) 4.5 (2006–2010) experiment, from 22 models, covering a 60-year period, denoted as ALL forcing (Table S1). Each model is re-gridded to the observed 1° × 1° grid.

#### CMIP5 pre-industrial runs

Pre-industrial control simulations (18,600 years, which provided 310 non-overlapping 60-yr blocks in total) from 35 models (Table S1) were used to estimate the characteristics of model unforced internal climate variability in order to increase number of independent noise data, which helps reduce sampling uncertainty in covariance estimation of the internal climate variability^38^.

#### CMIP3 20c3m and SRES runs

We utilise both 20c3m and SRES model experiments from the CMIP3 archive^30^. These include multiple 20c3m (1951–1999) and future greenhouse-gases under emission scenario of Special Report on Emissions Scenarios (SRES) A1B (2000–2010) experiments, from 7 models, covering a 60-year period. Each model is re-gridded to the observed 1° × 1° grid.

#### Individual forcing runs

Runs with and without particular radiative forcings were taken for eight CMIP5 models, and seven CMIP3 models. The experiments for CMIP5 examined include only natural forcings that include the effects of solar irradiance and volcanos (NAT), and experiments for CMIP3 examined include only anthropogenic forcings that include effects of greenhouse gases, sulphate aerosol, and ozone changes (ANT). We estimated the anthropogenic forced (ANT) signal for CMIP5 as the difference between ALL forcing and NAT forcing under the assumption of linearly additive responses to the external forcings. We estimated the NAT signal for CMIP3 as the difference between ALL forcing and ANT forcing under the assumption of linearly additive responses to the external forcings. Assuming that the effects of different forcings can cumulate linearly is commonly used when assessing individual forcing runs from climate models^16^. Therefore we continue this assumption and note that similar results are obtained for SST trends under ANT forcing estimated from the difference between ALL and NAT, or ANT-only forcing runs^39^. In addition, we assessed the likely effect of using different sets of models when calculating multi-model signals for the individual forcings. Overall, trends calculated using only the three CMIP5 models where all individual runs were available (i.e. bcc-cms1-1, CanESM2 and NorESM1-M; Table S1) are similar to that of using the complete set of CMIP5 models (Fig. S6).

### Detection method

To compare observed and modeled T_220m_ and T_14°C_ anomaly time series, an optimal fingerprinting technique^29^ was employed. Observations (**y**) were regressed onto multi-model mean response patterns (**X**, fingerprints of ANT, and NAT here) such that **y** = (**X − ν)β** + **ε**. Here regression coefficients **β** (or scaling factors) are obtained by the total least squares method, **ν** represents the component of **X** due to internal variability that remains after multi-model averaging, and **ε** represents residual variability that is generated internally in the climate system. The variance-covariance matrix of **ε** is estimated from pre-industrial control simulations, and that of **ν** is taken to be proportional to the variance-covariance matrix of **ε**, where the constant of proportionality reflects the methods used to calculate the multi-model ensemble response patterns. Two-signal analyses are undertaken whereby observations were regressed onto ANT and NAT simultaneously. This allows an examination of whether ANT and NAT are jointly detected and whether the influence of ANT is separable from that of NAT and internal variability in the observations. We divided 60-yr blocks of control simulations into two sets (150 blocks each) for the optimal fingerprinting analysis. The first set was used to obtain best estimates of **β** and the other set is used to estimate the 5–95% uncertainty range for **β** and also to carry out a standard residual consistency test^29^,^40^. This test offers a convenient method to check whether model-simulated internal variance is consistent with observational residual variance. Since the key aspect of this study was on long-term variability of upper-ocean changes plus short-lived variability due to volcanic aerosols, we calculated 2-year mean time series of anomalies to remove high frequency noise such as interannual variability. Therefore, 30-dimensional data vectors of observations and model simulations are obtained (from 60-year time series). Since data vectors have low dimension compared to the number of blocks of control simulations available for covariance matrix estimation (30 dimensions versus 150 blocks for each of the two covariance matrix estimates), we did not apply further dimension reduction such as empirical orthogonal function (EOF) truncations^40^, except for the five-point spatial model fingerprint analysis, for which we apply an EOF truncation of 30.

## Additional Information

**How to cite this article**: Weller, E. *et al*. Multi-model attribution of upper-ocean temperature changes using an isothermal approach. *Sci. Rep*. **6**, 26926; doi: 10.1038/srep26926 (2016).

## Supplementary Material

Supplementary Information
