# Supplementary material for: Multi-model attribution of upper-ocean temperature changes using an isothermal approach
Source: Sci Rep. 2016 Jun 1;6:26926. doi: 10.1038/srep26926 (PMC4887871; doi:10.1038/srep26926)
Supplement: Supplementary Information [file srep26926-s1.pdf]

*Supplementary Information*

# Multi-model attribution of upper-ocean temperature changes using an isothermal approach

Evan Weller[1]*, Seung-Ki Min[1]*, Matthew D. Palmer[2], Donghyun Lee[1], Bo Young Yim[3], Sang-Wook Yeh[3]

1. School of Environmental Science and Engineering, Pohang University of Science and Techonology, Pohang, Gyeongbuk, Korea
2. Met Office Hadley Centre, Exeter, UK
3. Hanyang University, ERICA, Korea

* Correspondence to evan.o.weller@gmail.com or skmin@postech.ac.kr

**Table S1. List of CMIP5 multi-model simulations used in this study**. Numbers represent ensemble size of ALL (natural plus anthropogenic forcing), NAT (solar and volcanic forcing) and CTL (unforced pre-industrial) simulations. See Methods for details.

| Model | ALL 1951-2010 [# of runs] | NAT 1951-2010 [# of runs] | CTL [# of 60-yr chunks] |
|---|---|---|---|
| ACCESS1-0 | | | 8 |
| ACCESS1-3 | | | 8 |
| bcc-csm1-1-m | 1 | | 6 |
| bcc-csm1-1 | 1 | 1 | 8 |
| BNU-ESM | 1 | | 8 |
| CanESM2 | 1 | 1 | 16 |
| CCSM4 | 1 | | 16 |
| CESM1-BGC | 1 | | 8 |
| CESM1-CAM5 | 1 | | 4 |
| CESM1-WACCM | | | 2 |
| CMCC-CMS | 1 | | 8 |
| CMCC-CM | 1 | | 4 |
| CNRM-CM5 | | 1 | 14 |
| CSIRO-Mk3-6-0 | | 1 | 8 |
| EC-EARTH | | | 6 |
| FGOALS-g2 | 1 | | 10 |
| FIO-ESM | | | 12 |
| GFDL-CM2.1 | 1 | | |
| GFDL-CM3 | 1 | | 8 |
| GFDL-ESM2G | 1 | | 8 |
| GFDL-ESM2M | 1 | | 8 |
| GISS-E2-H-CC | 1 | | 4 |
| GISS-E2-R-CC | 1 | | 4 |
| GISS-E2-H | | | 12 |
| GISS-E2-R | | | 14 |
| HadGEM2-CC | | | 4 |
| HadGEM2-ES | | 1 | 8 |
| IPSL-CM5A-LR | | 1 | 16 |
| IPSL-CM5A-MR | | 1 | 4 |
| IPSL-CM5B-LR | | | 4 |
| MIROC5 | 1 | | |
| MIROC-ESM-CHEM | 1 | | |
| MIROC-ESM | 1 | | |
| MPI-ESM-LR | | | 16 |
| MPI-ESM-MR | | | 16 |
| MPI-ESM-P | | | 18 |
| MRI-CGCM3 | 1 | | 8 |
| NorESM1-M | 1 | 1 | 8 |
| NorESM1-ME | 1 | | 4 |
| Total # of models<br># of runs | 22<br>22 | 8<br>8 | 35<br>310 |

**Table S2. Root-mean-square values for basin averaged time series**. Root-mean-square (RMS) value of observations and the CMIP5 ALL multi-model ensemble mean for $T_{220m}$ and $T_{14°C}$ for global and individual basin averages.

| | $T_{220m}$ | | $T_{14°C}$ | |
|---|---|---|---|---|
| **Basin** | **Obs** | **CMIP5** | **Obs** | **CMIP5** |
| Globe | 0.087 | 0.214 | 0.080 | 0.091 |
| Atlantic | 0.147 | 0.220 | 0.080 | 0.099 |
| Pacific | 0.078 | 0.191 | 0.094 | 0.095 |
| Indian | 0.106 | 0.250 | 0.084 | 0.089 |

**Table S3. Error reductions in model-observation time series comparison**. Root-mean-square (RMS) error of observations minus the CMIP5 ALL multi-model ensemble mean for $T_{220m}$ and $T_{14°C}$ for global and individual basin averages.

| **Basin** | **$T_{220m}$** | **$T_{14°C}$** |
|---|---|---|
| Globe | 0.090 | 0.050 |
| Atlantic | 0.076 | 0.055 |
| Pacific | 0.096 | 0.065 |
| Indian | 0.166 | 0.072 |

**Table S4. Comparison of trends in basin upper-ocean temperatures in the observations with and without the influence of the leading mode of the Pacific multidecadal variability.** Observational trend slopes with ($S_{OBS}$) and without the influence of the Pacific Decadal Oscillation ($S_{OBS*}$) are given for comparison over the period 1951-2010. Units for °C per 60 years.

| | **$T_{220m}$** | **$T_{14°C}$** |
|---|---|---|
| **Basin** | **$S_{OBS}$<br>$S_{OBS*}$** | **$S_{OBS}$<br>$S_{OBS*}$** |
| Globe | 0.22<br>0.20* | 0.22<br>0.19* |
| Atlantic | 0.41<br>0.36* | 0.23<br>0.16* |
| Pacific | 0.14<br>0.14* | 0.22<br>0.18* |
| Indian | 0.11<br>0.07* | 0.19<br>0.12* |

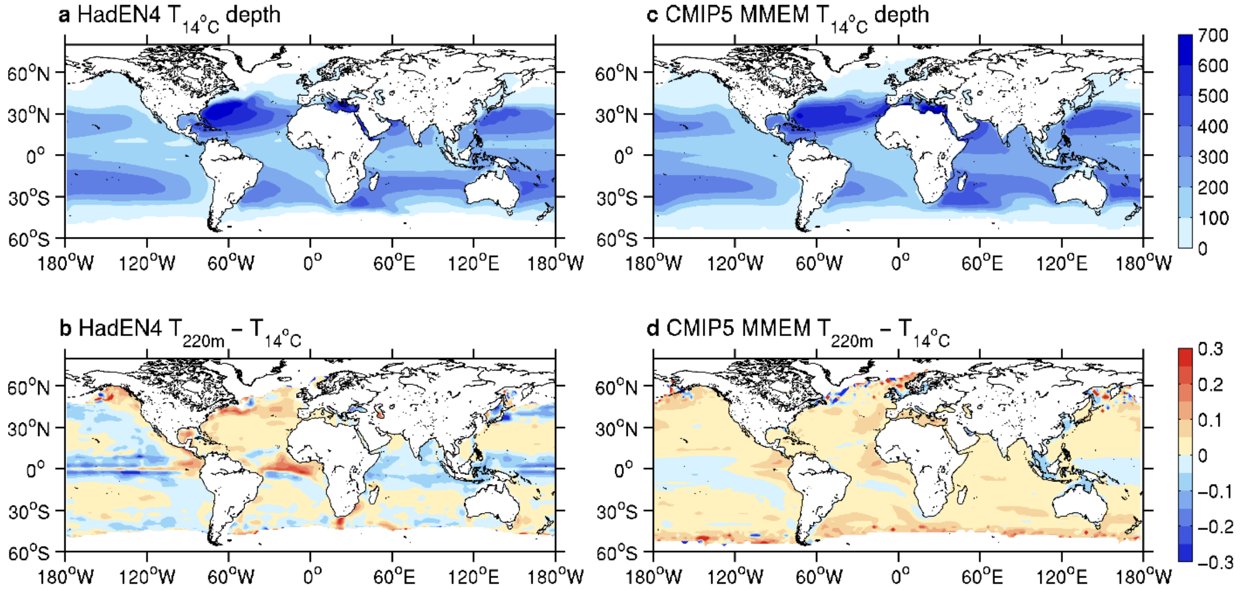


**Figure S1 | Spatial maps of the mean depth of the 14°C isotherm and differences in $T_{220m}$ and $T_{14°C}$ trends of observations and simulations**. (a), Climatological (1971-2000) mean depth of the 14°C isotherm from HadEN4. (b), Difference in trends (°C decade$^{-1}$) of the average temperature anomaly above 220 m ($T_{220m}$) and the average temperature anomaly above the 14°C isotherm ($T_{14°C}$) from HadEN4 over 1951-2010. (c), (d), The same as (a) and (b) but for the from the CMIP5 multi-model ensemble mean. All maps were produced using licensed MATLAB (release R2015B available at http://au.mathworks.com/products/matlab/index.html).

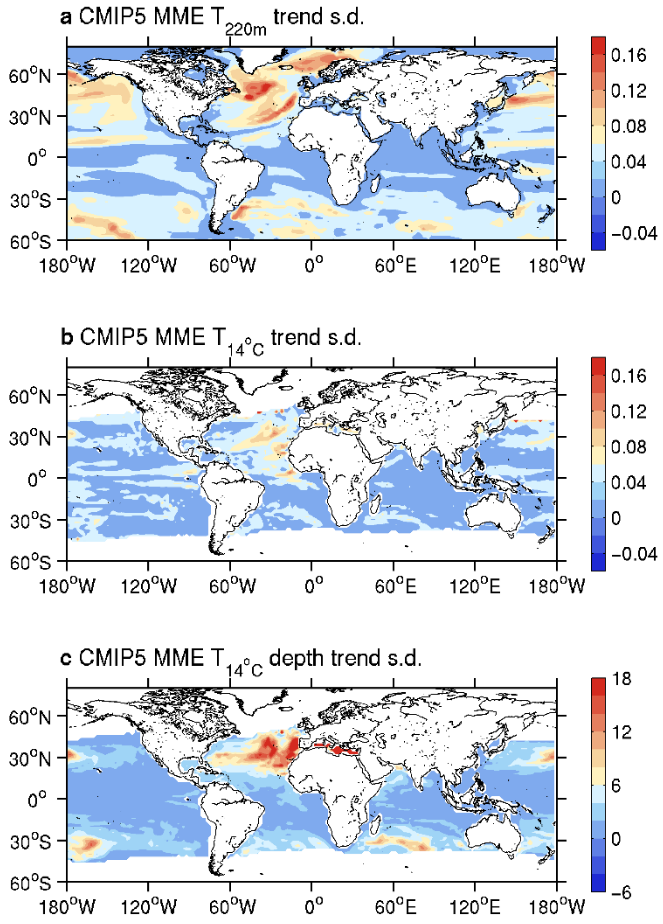


**Figure S2 | Spread of the simulated upper-ocean temperatures and 14°C isotherm depth trends**. (a), Standard deviation (in °C) of the multi-model ensemble $T_{220m}$ trends. (b), The same as (a) but for $T_{14°C}$. (c), The same as (a) but for $D_{14°C}$ (in m). All maps were produced using licensed MATLAB (release R2015B available at http://au.mathworks.com/products/matlab/index.html).

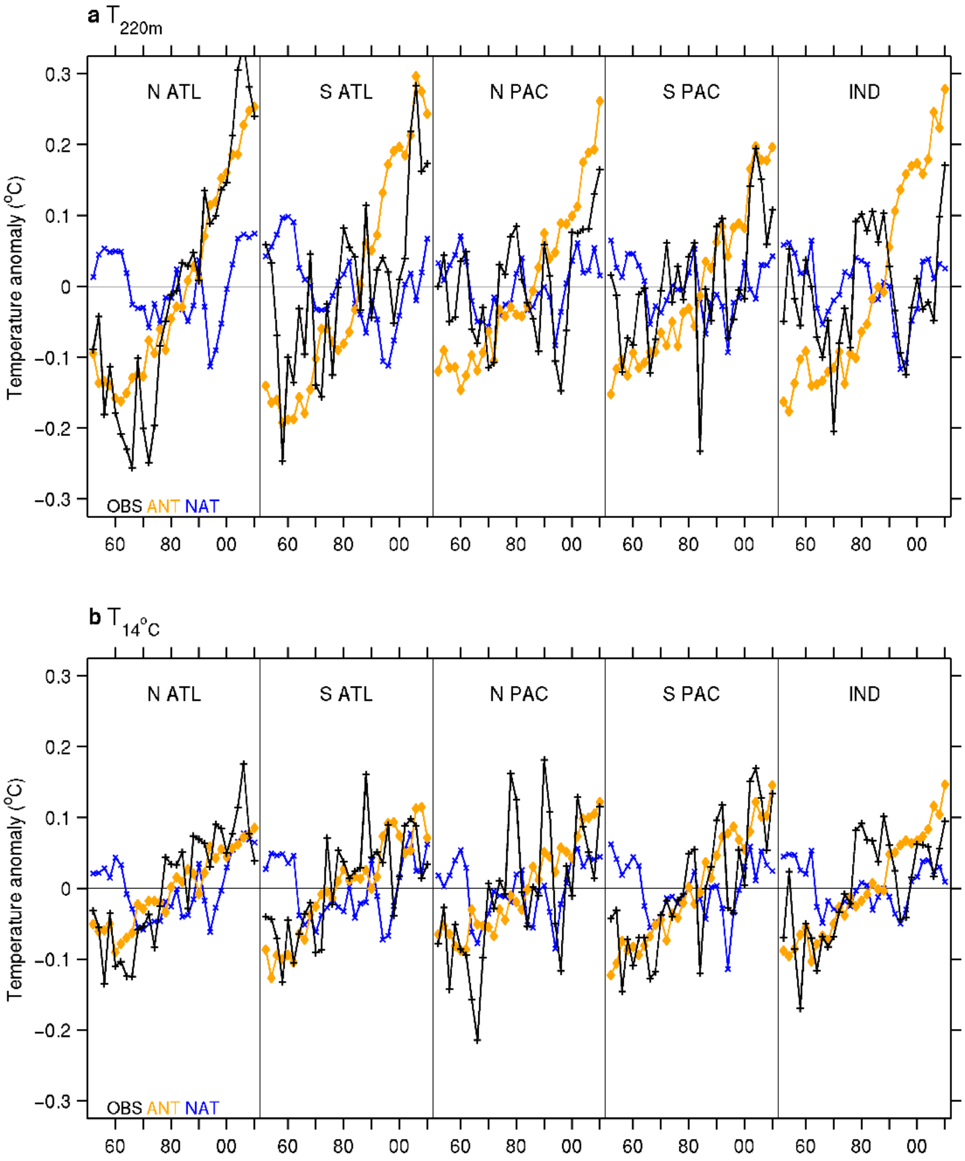


**Figure S3 | Observed and simulated upper-ocean temperature anomalies averaged over the oceans basins**. (a) 2-year mean average ocean temperature anomalies above 220 m ($T_{220m}$) for the five different ocean basins, North Atlantic, South Atlantic, North Pacific, South Pacific, and the Indian Ocean. HadEN4 (black) is compared to the CMIP5 multi-model ensemble mean response for anthropogenic (ANT, estimated as ALL minus NAT, orange) and natural (NAT, blue) forcing simulations. (b), The same as (a) but for 2-year mean average temperature anomalies above the 14°C isotherm ($T_{14°C}$).

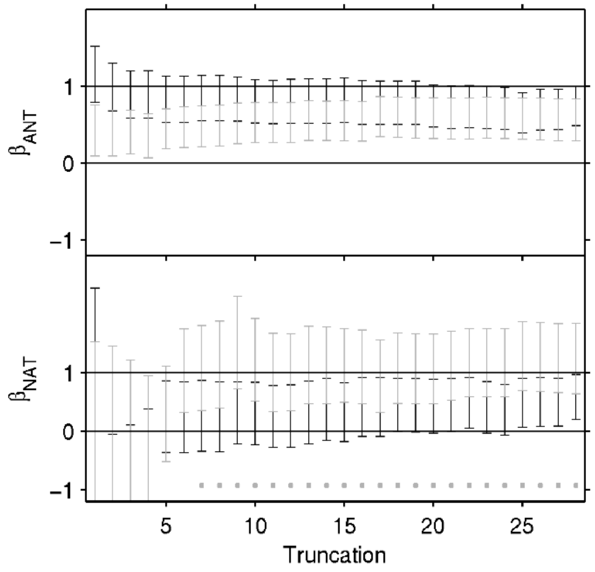


**Figure S4 | Results from optimal detection analysis of upper-ocean temperatures anomalies**. The estimated scaling factors for EOF truncation values up to 30 for two-signals of anthropogenic (ANT, top) and natural (NAT, bottom) forcings by which the simulated 2-year averaged $T_{220m}$ (gray) and $T_{14°C}$ (black) responses in the oceans should be multiplied to best match the observations over 1951-2010. Gray and black squares under scaling factors indicate that the residual variability remaining in the observations after removing the scaled response is inconsistent with model internal variability in $T_{220m}$ and $T_{14°C}$, respectively.

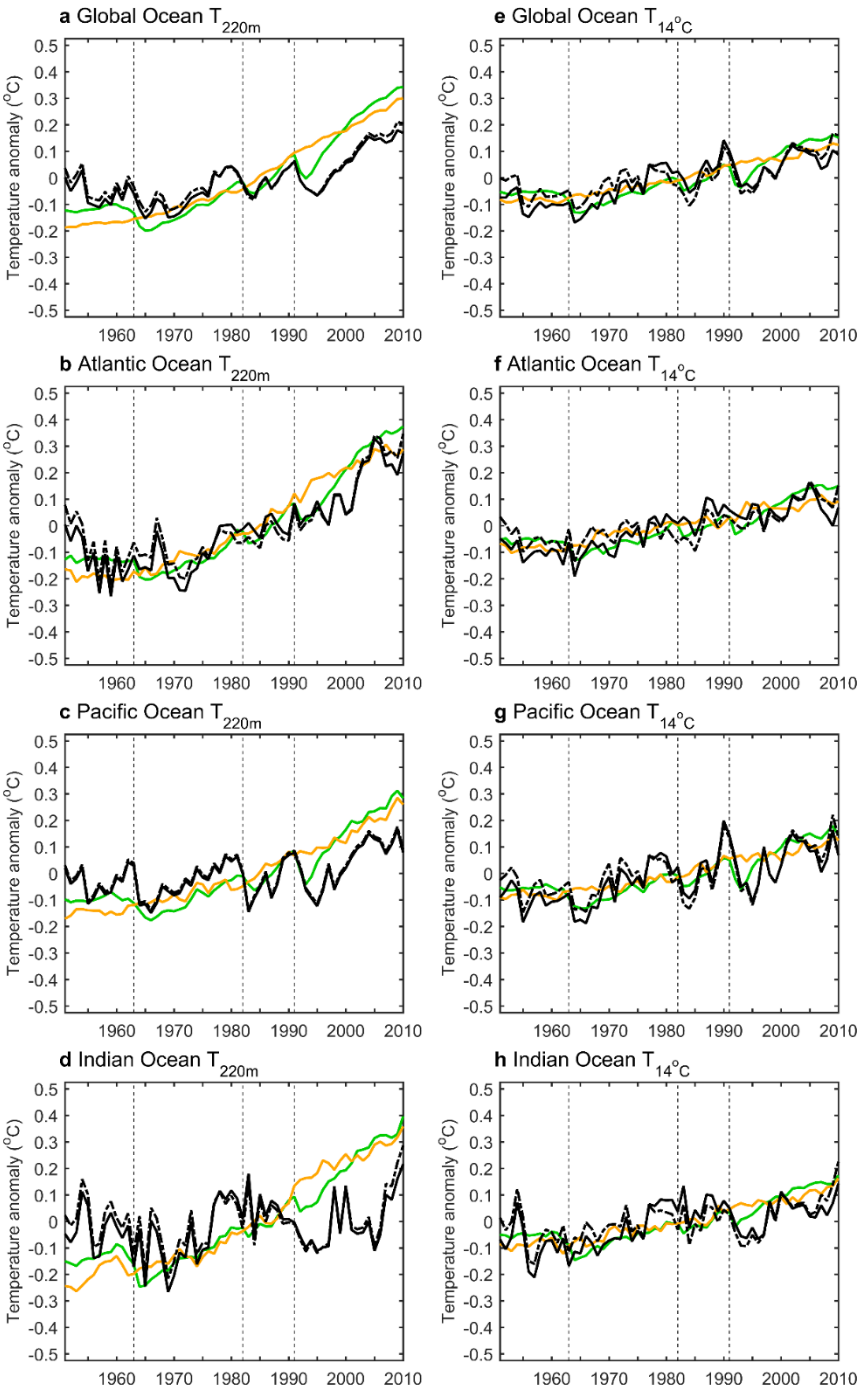


**Figure S5 | Observed and simulated upper-ocean temperature anomalies averaged over the oceans basins**. The same as Figure 3 in the main text except comparing the observed time series with (solid black) and without (dashed black) the influence of the Pacific Decadal Oscillation (removed via linear regression).

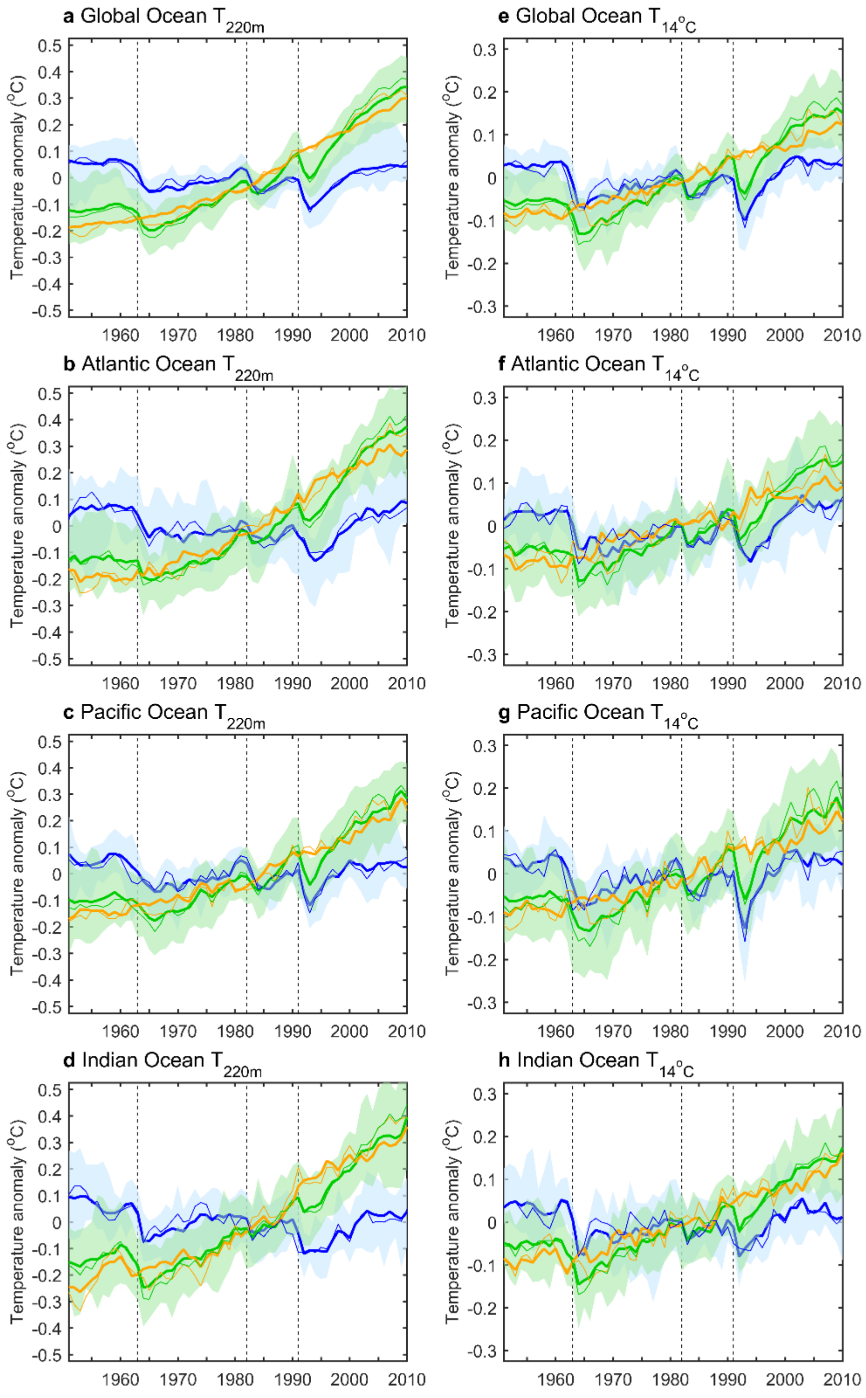


**Figure S6 | Observed and simulated upper-ocean temperature anomalies averaged over the oceans basins**. The same as Figure 3 in the main text except comparing the time series using all the CMIP5 multi-model ensemble (thick lines) and only the three models where both ALL and NAT forcing runs are available (thin lines; Table S1).
